# Editorial

**Published:** 1869-07

**Authors:** 


### Editorial.

Chicago Medical Society.—The regular meetings of this Society are now being held once in two weeks. The Secretary, Dr. H. Wanzer, has kindly furnished us with notes of the proceedings of the meetings during the past month, but so much of our space has been occupied with the proceedings of the Illinois State Medical Society and the Iowa State Society, that we must omit their insertion, at least, for the present.

---

Summer Medical Institute.—The Spring and Summer courses of instruction in both the Chicago Medical College and the Rush Medical College, closed with the 1st of July. Excellent classes of young men have been in attendance on the courses in both schools, and their opportunities for improvement have been as good, especially in the Clinical Department, as could be obtained in any city in the Union. The Announcements for the coming year, and an account of the new Mercy Hospital building will be given in our next issue.

---

VAGINISMUS.—Sometime last year, we published a case of this affection, in which the parties had been married three years without accomplishing sexual intercourse. We operated on the case as recommended by J. MARION SIMS. The result was entirely satisfactory; and a day or two since the wife became the mother of a full-sized, healthy boy, much to the joy of both parents.

---

### KENTUCKY SCHOOL OF MEDICINE.

---

LOUISVILLE, KY., *May 20th, 1869.*

At a meeting of the Faculty of this School, May 13th, the following preamble and resolution were unanimously adopted:

WHEREAS, The medical colleges of the United States have, hitherto, in vain sought for an expression of the wish of the American Medical Profession in regard to the amount of fees proper for a single official course of collegiate lectures; and

WHEREAS, The American Medical Association did, by a vote conspicuously unanimous, decide on the 6th of May, 1869, that $120 should be accepted as the MINIMUM amount to be charged for such a course of lectures; and

WHEREAS, This vote is morally and honorably binding upon all who personally or by delegated authorlty entered into it; and

WHEREAS, The Faculty of the Kentucky School of Medicine, in common with all reputable members of the Medical Profession, accepts the decisions of the American Medical Association, as representing, through its delegated organizations, the sentiment and desire of the great body of American physicians;

*Be it hereby Resolved,* That the Feculty of the Kentucky School of Medicine acknowledges this officially expressed wish of the Association as its guide in this relation, and that it will hereafter charge $120 for each official course of its collegiate lectures. L. J. FRAZEE, M.D., *Dean.*

The above circular from the Kentucky School of Medicine is excellent as far as it goes. We wish every Medical College in the country would thus officially recognize the authority of the American Medical Association. It would afford a good prospect for a speedy improvement in the whole system of medical education. That Association, at its first meeting, more than twenty years since, voted with "conspicuous unanimity" that the length of the official College Lecture Terms should be *six months;* that a *standard of preliminary education* should be required; and it has repeated these votes scarcely less than a dozen of times since. Again, at a full meeting in 1866, the same Association voted with entire unanimity, not only, in favor of a Lecture Term of *six months*, and a standard of *preliminary education*, but also in favor of *three courses of Lectures*, and a consecutive order by dividing the branches of study and the students into *three classes, etc.* Will the Kentucky School of Medicine, and all other schools, as promptly acknowledge and adopt all these important recommendations of the Association as they have the one concerning *fees?* Most certainly, if the National organization is competent to decide what should be the *minimum fee* for an official Lecture Term, it must be equally competent to decide what should be the *minimum length* of such Lecture Term, and *how many* such should be attended to render the student elligible to graduation. As has been well said by the Editor of the Leavenworth *Medical Herald*, there are other methods of underbidding for students more injurious to the profession than simply lowering the nominal lecture fees. The giving of credit for lecture fees; the adoption of short terms; few examinations; no positive requisition for clinical attendance or practical dissections, are all means for attracting students and cheapening the Diploma, far more injurious in their practical results than an openly adopted low rate of fees. Indeed, there can be no uniform standard or rate of Lecture fees in our Medical Colleges, until we have secured first uniformity in the plan of their organization, their curriculem of studies, the length of their Lecture terms, and the number of such terms to be attended before graduation. The only true test of the "*cheapness*" of a Medical School, is the aggregate expense the student is required to incur before obtaining his Diploma. For instance, if the Kentucky School of Medicine should charge a Lecture fee of $120 00, and require the student to attend two courses of four months each before graduation, the student's aggragate expenses would stand thus:

| | |
|---|---|
| Lecture fees, two courses, $120 00 each, | $240 00 |
| Two courses of four months each, 35 weeks, board $5 00 per week, | 175 00 |
| Total, | $415 00 |

If the Chicago Medical College should charge a Lecture fee of only $50 00, and require attendance on *three* courses of Lectures of six months each before graduating, the student would foot up his aggregate expenses thus:

| | |
|---|---|
| Lecture fees, three courses, $50 00 each, | $150 00 |
| Three courses of six months each, 78 weeks board at $5 00 per week, | 390 00 |
| Total, | $540 00 |

Would the student have any difficulty in determining which was the *cheap* school? We are in favor of a fair and reasonable rate of Lecture fees in all Medical Colleges. We are also in favor of making them as nearly uniform as practicable. And the foregoing remarks and illustrations have been made for the sole purpose of showing that to attain these ends requires a consideration and adjustment of the whole system of Medical College organizations throughout the country.

---

DR. ALDEN MARCH.—Another of the noble and generous spirits in our profession has taken its departure. Dr. ALDEN MARCH, of Albany, New York, died June 17th, 1869, at the age of 74 years. He had practiced medicine in Albany 49 years; was the founder of the Albany Medical College, and the City Hospital, and one of the most eminent practitioners and teachers of Surgery in our country. He was known, respected, and honored by the profession on both sides of the Atlantic. Only a short time since he had donated his valuable pathological collection to the Museum of the College, with a legacy of $1,000, the interest on which was to keep the Museum in order, and a like sum to the City Hospital, to keep the same supplied with instruments. He was a man of great energy and perseverance, of high social qualities, and a devoted Christian. Although at the ripe age of 74 years, he died in the midst of his professional activity and usefulness. Only little more than six weeks previous to his death he was in attendance on the Annual Meeting of the American Medical Association, in New Orleans, one of its most honored ex-presidents; and was congratulated by his friends on account of his apparent health and vigor. But so it is; in the midst of life the *grim messenger* is often at the door.

---

LIBRARY MEDICAL DEPARTMENT UNIVERSITY OF LOUISVILLE,
*May 27th*, 1869.

At a meeting of Delegates from Medical Colleges for the purpose of considering the question of fees, which was held this day, the following Colleges were represented by Delegate or letter, *viz:*

University of Nashville; Shelby Medical College, Nashville; Memphis Medical College; St. Louis Medical College; Humbolt Medical College, of St. Louis; Rush Medical College, of Chicago; Chicago Medical College; Indiana Medical College, of Indianapolis; Miami Medical College of Cincinnati; University of Louisville.

On motion, Dr. Bowling was elected Chairman, and Dr. Bayless, Secretary of the meeting. After a prolonged conference, the following preamble and resolutions were adopted:

WHEREAS, The call for a Convention of Delegates from the Medical Colleges of the West, for the purpose of arranging a uniform scale of fees, sent by the Faculty of the Medical Department of the University of Louisville to the Colleges of Nashville, Memphis, Cincinnati, Columbus, Cleveland, Detroit, Chicago, St. Louis, Indianapolis, and Louisville, has met with a cordial response in person or by letter from a majority of said Colleges; and

WHEREAS, The fact that several of said Colleges have issued their Announcements for the ensuing sessions, makes definite action at present impossible; and

WHEREAS, The views and opinions of the various schools as given by delegates and letters differ greatly; therefore be it

*Resolved*, That it is the hope of this Convention that a uniform scale of charges shall be adopted by all the Medical Colleges of our country, and we do most earnestly advise such a scale as shall be agreed upon; and it is our belief that the glory and usefulness of our profession would be enhanced by the adoption of the highest rate advised by the American Medical Association.

*Resolved*, It is not less to be hoped that all the Medical Colleges of our country would fix a higher standard of preliminary and medical education as a pre-requisite for graduation.

*Resolved*, That the Convention request all the Medical Colleges in the United States to send each one Delegate to a meeting to be held in Washington, on Monday, May 2d, 1870, to take efficient steps toward carrying out in good faith the recommendations of the American Medical Association in reference to medical education, and also to form a permanent association of American medical teachers.

*Resolved*, That a copy of these proceedings be sent to all the medical journals in the country.

WM. K. BOWLING, *President.*

GEO. W. BAYLESS, *Secretary.*

---

TREATMENT OF LEAD POISONING.—Dr. Wm. Frank-Smith states (*Lancet*) that in lead-poisoning the treatment by iodide of potassium has been less efficacious in his practice than by sulphuric acid and the sulphates. The formula used by him, which acts, he says, "with remarkable celerity and certainty," is as follows:—Sulphate of quinine, sulphate of iron, of each, one grain; strychnia, $\frac{1}{36}$ of a grain; dilute sulphuric acid, five minims; sulphate of magnesia, one drachm; water, one ounce: three times a day.—*N. Y. Med. Gazette.*

---

PARALYSIS FOLLOWING THE USE OF CHLOROFORM.—At the session of the k. k. Gesellsch. d. Aerzte, Vienna, Feb. 19, there was reported a case in which the patient became completely aphonic and aphasic after awaking from the slight degree of narcosis required for the removal of a tooth. There was also at that time considerable spasm of the glottis, most marked during inspiration. The loss of voice and speech continued during five weeks; but for three weeks past, the patient has been able to speak in a low voice. The treatment up to the date of report was mainly by electricity; the constant stream is found most useful. Dr. Hofmokl, who reported the case, believes that the symptoms were due to cerebral apoplexy, caused by the chloroform narcosis. The patient was a perfectly healthy woman, a cook, aged 22, and never had hysteria.—

*Wochenbl. d. k. k. Ges. d. A. in W., Mar. 3.—Boston Med. and Surg. Journal.*

---

At the meeting of the Lyceum of Natural History, on Monday last, Professor Edwards brought forward a useful application of the spectroscope to the pharmaceutist's business. He had noticed that druggists had put chemicals which were affected by sunlight in blue bottles—the very worst color to use, as it permitted nearly all the chemical rays to pass through. At his suggestion, yellow bottles had been substituted by a member of his class; this color stopping all the chemical rays. The question was, to determine the exact tint of yellow to use. By means of the spectroscope, in a few seconds he had been able to pick out the best glass to make the bottles of, as the spectrum was more shortened by some than by others.—*N. Y. Med. Gazette.*

---

Cure of Cataract without Operation.—Dr. Travignor has communicated to the Paris Academy of Medicine a method by which cataract may be cured without an operation. It consists in applying to the diseased eye a phosphureted collyrium, which operates by gradually restoring the transparency of the crystalline lens, from the circumference to the centre, which is the last to yield. The formula is as follows:—Oil of sweet almonds, 30 grammes; phosphorus, 10 centigrammes; dissolve in a water bath at 80 deg. centigrade, in a close and full vessel. Three or four times a day, 4 grammes of this solution should be instilled between the eyelids of the diseased organ, continuing to do so for several months.—*The Med. Mirror and Med. Record.—N. Y. Med. Gazette.*

---

Mortality in the Hotel Dieu.—Vicq. d' Az yr and Lavoisier having been requested by the Academy of Sciences of Paris to compare with respect to the relative mortality the Hotel Dieu and La Charite, have signed a report in which the following passage occurs: "The Hotel Dieu, in fifty-two years, out of 1,008,741 patients has lost 244,720, a proportion of one in four and a-half. La Charite, which has only had a mortality of one in seven and a-half, has lost 168,700. From these statistics results the fearful consideration that the Hotel Dieu has in fifty-two years cost France 99,044 citizens, whose lives would have been preserved if the Hotel Dieu had enjoyed as favorable position as that of La Charite. The loss of life in these fifty-two years represent 1906 deaths a-year, about the tenth part of the total and continuous mortality of Paris. The maintenance of this hospital, or rather of the situation which it occupies, produces therefore the same effect as would a plague which unceasingly desolated the capital.

---

VAGINITIS.—Dr. Neftel, of New York, relates three cases of vaginitis occurring in women of high social position; in one of whom, on account of the aggravated form of the disease, sexual intercourse had become impossible, coincident with a saturnine intoxication, resulting from the prolonged use of a cosmetic containing lead. The intoxication manifested itself by paralysis and atrophy of the extensors, so that the patients were unable to extend their hands, fingers, or thumbs, while the supinator muscles, as well as the deltoid, biceps, and triceps, were in a normal state. The electro-muscular contractility and sensibility were very much diminished, if not entirely abolished. Two of the patients were cured by the internal use of the iodide of potassium and sulphur, and without having employed any local means for the vaginitis. It disappeared at the same time with the paralysis, so that the married woman who had hitherto been sterile, and had for that cause consulted Dr. Marion Sims, has since become a mother.—*L' Union Medicale and Am. Jour. of Obstetrics.—N. Y. Med. Gazette.*

---

ON CERTAIN PHYSICAL AND PHYSIOLOGICAL PROPERTIES OF MUSCLES.—M. Chmoulevitch has just published, on the above question, an interesting paper, which may be summed up thus: —1. When heat acts upon a muscle at rest, it produces two effects—one purely physical the other physiological. The physical effect is observed only between 2 and 28 degrees centigrade. Between these limits, the muscle acts, under the influence of heat, in a manner opposed to all other known bodies: it shortens in becoming warm, and lengthens in cooling. 2. Beyond 28 degrees centigrade, the purely physical influence which has just been described is complicated by a novel action of a physiological nature. If the temperature of the muscle be raised in a regular manner up to 40 or 41 degrees, a shortening is produced, of which the rapidity increases between 35 and 41 degrees; the muscle is in a condition which has been termed *rigidity through heat.* 3. When it is required to produce this kind of rigidity in two muscles, one of which has been separated from the body two or three hours before the other, a higher degree of temperature must be employed for the first muscle. 4. The size of the muscle diminishes during cadaveric rigidity. 5. The specific weight of the muscle increases under the same influence. 6. At the same time, the absolute weight of the muscle diminishes. 7. The size of the muscle diminishes in like manner during *rigidity through heat.* 8. The mechanical tension of the muscles also produces a diminution of their size. This result, according to the mechanical theory of heat, is in accordance with another fact, which is—9. That in muscles (and especially living muscles) a certain quantity of heat is set free under the influence of mechanical tension.—*Lancet.—N. Y. Med. Gazette.*

---

Saccharite of Iron as a Medicine.—Oxide of iron forms an important constituent of the blood; it gives the red color to blood, and assists in the building up of the corpuscles. Liebig is of the opinion that the oxide of iron plays a very important part in the oxidation of the food, and in keeping up the heat of the body. If from the blood, by rapid growth, or any cause, iron is removed, the normal influence of the blood on the nerves is changed, and all of the symptoms of chlorosis are manifest. According to the physiological researches of Mialhe, Bouchartd, and Quevenne, in Paris, iron in a soluble condition is much more readily assimilated than when it is in the insoluble modification; it is also much more easily digested when taken with food than upon an empty stomach; and as the total amount the blood is able to assimilate is very small, the object can only be reached by continuing the use of it for a considerable time.

It has been found that the hydrated sesqui oxide of iron is soluble in rock candy—from this solution ammonia will no longer precipitate the oxide. Advantage has been taken of this reaction in Germany, and sugar bon-bons are now prepared under the name of Dr. E. Fleischer's soluble iron saccharate. They are put up like sugar plums, and contain $\frac{1}{15}$ of a grain of metallic iron in each bon-bon. They are also flavored with some kind of *liqueur*, and would never be suspected to be medicine by any child. Beside the bon-bons, the solution is kept in bottles, in a form suitable for drinking with food. The pyrophosphate of iron is also prepared in a similar way, thus affording phosphorus and iron at the same time. This preparation of iron has not been introduced in the United States, and ought to be better known.—*Journal of Applied Chemistry.—N. Y. Med. Gazette.*

### Mortality Report for Month of April, 1869:—

| | | | | | |
|---|---|---|---|---|---|
| Accidents, falls, | 6 | Chicken-pox, | 1 | Kidneys, Bright's dis- | |
| " drowned, | 1 | Croup, membranous, | 1 | ease, | 3 |
| " amputation | | Debility, | 2 | " paralysis of, | 1 |
| for injuries, | 1 | " general, | 1 | " tumor of, | 1 |
| " internal in- | | Diabetes, | 1 | Liver, cirrhosis of, | 1 |
| juries, | 1 | Diarrhœa, | 3 | Lungs, congestion of, | 2 |
| " injury to | | " chronic, | 2 | " paralysis of, | 1 |
| brain, | 1 | Diphtheria, | 5 | Measles, | 1 |
| " run over by | | " and teething, | 1 | Meningitis, | 6 |
| wagon, | 1 | Dropsy, | 3 | " and brain | |
| " circular saw | 1 | Dysentry, | 2 | effusion, | 1 |
| " kick of horse | 1 | Emphysema, | 1 | " cerebro-spi- | |
| " poisoning, | 1 | Encephalitis, | 4 | nal, | 3 |
| " overdose | | " and meningitis, | 1 | " tubercular, | 1 |
| wine colch- | | Endo-carditis, | 1 | Metritis, | 1 |
| icum, | 1 | Erysipelas, | 2 | " and gastritis, | 1 |
| " railroad, | 2 | Fever, congestive, | 1 | Manslaughter, | 1 |
| " crushing of | | " puerperal, | 3 | Miscarriage, | 1 |
| both legs, | 1 | " remittent, | 1 | Old age, | 8 |
| Abscess, hepatic, | 1 | " scarlet, | 44 | Œsophagitis, inflam- | |
| Anus, imperforate, | 1 | " malignant, | 1 | mation of, | 1 |
| Angina tonsillaris, | 1 | " and meningitis, | 1 | Œdema pulmonem, | 2 |
| Apoplexy, | 5 | " typhoid, | 6 | Paralysis, | 2 |
| Ascitis, | 1 | " typhoid and | | Peritonitis, | 5 |
| Arachnitis, | 1 | bronchitis, | 1 | Phlebitis, uterine, | 1 |
| Births, still, | 35 | Gangrene of leg, | 1 | Pneumonia, | 28 |
| " premature, | 19 | Glands, prostate, in- | | " broncho, | 3 |
| " tedious, | 2 | flammation of, | 1 | " typhoid, | 1 |
| Brain, congestion of | 5 | " of neck " | 1 | " whooping- | |
| " and lungs " | 1 | Gastritis, | 4 | cough, | 1 |
| disease " | 1 | " acute, | 1 | Pyæmia, | 1 |
| " injury to, | 1 | Hemorrhage, internal, | 1 | Rheumatism and car- | |
| " inflammation of, | 3 | Heart, disease of, | 4 | diac disease, | 1 |
| Bronchitis, | 3 | " and liver, " | 1 | Scrofula, | 2 |
| Cancer, | 2 | " hypertrophy of, | 2 | Suicide, | 5 |
| Cerebritis, | 1 | " organic disease of, | 2 | Small-pox, | 2 |
| " and soften- | | " rheumatism of, | 1 | Spine, disease of, | 1 |
| ing of brain, | 1 | " valvular disease of, | 1 | Suffocation, | 1 |
| Childbirth, | 2 | Hepatitis, acute, | 1 | Tabes mesentrica, | 6 |
| Chorea and convulsions | 1 | " chronic, | 1 | Teething, | 2 |
| Convulsions, | 30 | Hernia, | 1 | " and convulsions, | 1 |
| " puerperal, | 1 | Hydrothorax, | 1 | Whooping-cough, | 10 |
| Colic, | 1 | Hydrocephalus, | 4 | Unknown, | 1 |
| Consumption, | 53 | " acute, | 2 | | |
| " and emphysema, | 1 | Inanition, | 8 | | 380 |
| Croup, | 5 | Intemperance, | 3 | | |

### COMPARISON.

Deaths in April, 1869, 380 | Deaths in April, 1868, 305 | Increase, 75
Deaths in March, 1869, 353 | Increase, 27

### AGES.

| | | | | | |
|---|---|---|---|---|---|
| Under 1 | 91 | 30 to 40 | 38 | 80 to 90 | 1 |
| 1 to 3 | 70 | 40 to 50 | 23 | 90 to 100 | 1 |
| 3 to 5 | 30 | 50 to 60 | 22 | Unknown | 2 |
| 5 to 10 | 19 | 60 to 70 | 11 | | |
| 10 to 20 | 16 | 70 to 80 | 14 | Total, | 380 |
| 20 to 30 | 41 | | | | |

| | | | | | |
|---|---|---|---|---|---|
| Males, | 199 | Females, | 181 | Total, | 380 |
| Single, | 271 | Married | 109 | Total, | 380 |
| White, | 374 | Colored, | 6 | Total, | 380 |

NATIVITY.

| | | | | | |
|---|---|---|---|---|---|
| Atlantic Ocean, | 2 | Denmark, | 1 | Norway, | 6 |
| Belgium, | 1 | England, | 5 | New Brunswick, | 1 |
| Bohemia, | 5 | France, | 2 | Sweden, | 7 |
| Canada, | 4 | Germany, | 55 | Scotland, | 9 |
| Chicago, Native, | 48 | Holland, | 3 | Unknown, | 3 |
| Chicago, Foreign, | 123 | Ireland, | 39 | | |
| U. S., other parts, | 65 | Isle of Man, | 1 | Total, | 380 |

MORTALITY BY WARDS FOR THE MONTH.

| Ward. | Mortality. | Pop. in 1868. | One death in | Ward. | Mortality. | Pop. in 1868. | One death in |
|---|---|---|---|---|---|---|---|
| 1 | 6 | 9,094 | 1,515⅔ | 14 | 28 | 14,839 | 530 |
| 2 | 4 | 13,074 | 3,268½ | 15 | 26 | 21,078 | 810 2-5 |
| 3 | 15 | 15,076 | 1,005 1-15 | 16 | 24 | 15,465 | 644⅓ |
| 4 | 27 | 17,796 | 655 2-5 | County hos. | 9 | | |
| 5 | 24 | 16,033 | 668 | Accidents, | 18 | | |
| 6 | 15 | 13,083 | 872 | Mercy Hosp. | 3 | | |
| 7 | 37 | 25,492 | 689 | Suicides, | 5 | | |
| 8 | 21 | 15,813 | 753 | St. Jo. Orph. Asylum | 6 | | |
| 9 | 24 | 19,297¼ | 804 | Manslaughter | 1 | | |
| 10 | 13 | 12,925 | 994 | Lake Hosp. | 1 | | |
| 11 | 18 | 14,340 | 796⅔ | Home for the Friendless, | 6 | St. Luke's Orph. Asylum, | 1 |
| 12 | 31 | 17,485 | 564 | | | | |
| 13 | 17 | 11,164 | 656⅔ | | | | |

Total, 380

### Mortality for the Month of May, 1869:—

| | |
|---|---|
| Accidents, brain, concussion of | 1 |
| " drowned, | 3 |
| " burns, | 1 |
| " by laudenum | 1 |
| " overdose whiskey, child, by self | 1 |
| " lightening, | 1 |
| " run over by engine, | 2 |
| " railroad cars | 1 |
| " pistol shot, | 1 |
| " by fall, | 1 |
| " injury to spine, | 1 |
| " fracture of spine, | 1 |
| Abscess, hepatic, | 1 |
| Anasarca, | 1 |
| Angina, | 2 |
| Apoplexy, | 4 |
| " from intemperance, | 1 |
| Ascitis, | 2 |
| Births, premature, | 39 |
| " still, | 10 |
| Bowels, inflammation, | 2 |
| Brain, congestion of, | 2 |
| " inflammation of | 1 |
| Bronchitis, | 3 |
| " capillary, | 1 |
| Cancer encephaloid, | 1 |
| Cerebritis, | 1 |
| Cerebritis and paralysis | 1 |
| Chest, dropsy of, | 1 |
| Cholera infantum, | 2 |
| Convulsions, | 34 |
| " puerperal, | 1 |
| Consumption, | 44 |
| " caries of pelvis bones, | 1 |
| Congestion, visceral, | 1 |
| Croup, | 4 |
| " membranous, | 1 |
| Cyanosis, | 1 |
| Cyanch maligna, | 1 |
| Debility, | 1 |
| " general, | 5 |
| Deficient development, | 2 |
| Diarrhœa, | 6 |
| " chronic, | 6 |
| " convulsions | 1 |
| Diphtheria, | 2 |
| " scarlet fever, | 1 |
| Drowned, | 2 |
| Dropsy and pneumonia | 1 |
| Dysentery, | 3 |
| Encephalitis, | 1 |
| Enteritis, | 3 |
| Fever, congestive, | 1 |
| " puerperal, | 4 |
| " scarlet, | 26 |
| " " and pneumonia, | 1 |
| " typhoid, | 13 |
| Hemorrhage of liver, | 1 |
| Gangrene, from injuries, | 1 |
| Heart, disease of, | 5 |
| " rheumatism of, | 1 |
| " valvular dis. of, | 1 |

| | |
|---|---|
| Hernia, strangulated, | 3 |
| Hydrothorax, | 1 |
| Hydrocephalus, | 10 |
| " acute, | 4 |
| Inanition, | 8 |
| Intemperance and exposure, | 2 |
| " and childbirth | 1 |
| Intupusceptio, | 1 |
| Jaundice, | 2 |
| Kidneys, Bright's disease of, | 5 |
| Liver, cancer of, | 2 |
| " fatty degeneration of, | 1 |
| " organic disease, | 1 |
| " abscess of, | 1 |
| Lungs, congestion of, | 5 |
| " emphysema of, | 1 |
| Lungs, paralysis of, | 1 |
| Leucothemia following childbirth, | 1 |
| Lungs, hemorrhage of, | 1 |
| Mouth, canker-sore, | 3 |
| Manslaughter, | 1 |
| Measles, | 9 |
| Meningitis, | 3 |
| " cerebro-spinal, | 3 |
| " tubercular, | 1 |
| Old age, | 8 |
| Œdema pulmonum, | 2 |
| Paralysis, | 2 |
| Peritonitis, | 3 |
| " puerperal, | 2 |
| Pleurisy, | 1 |
| Pneumonia, | 14 |
| " typhoid, | 5 |
| Shock from excessive whipping, | 1 |
| Small-pox, | 3 |
| Shock from surgical operation, | 1 |
| Stomach and liver, disease of, | 1 |
| " cancer of, | 1 |
| " organic disease | 1 |
| Suicide, | 1 |
| Tabes mesentrica, | 12 |
| Teething, | 4 |
| " convulsions, | 3 |
| Tetanus, | 2 |
| Uterus, cancer of, | 3 |
| " disease of, | 1 |
| Whooping-cough, | 11 |
| " pneumonia, | 2 |
| Total, | 372 |

COMPARISON.

Deaths in May, 1869,-- 372 | Corresponding month, 1868,-- 321 | Increase,- 51

Deaths in preceding month, 1869,-- 381 | Decrease, -------------------- 9

AGES.

| | |
|---|---|
| Under 1 year | 95 |
| 1 to 3 | 69 |
| 3 to 5 | 26 |
| 5 to 10 | 21 |
| 10 to 20 | 17 |
| 20 to 30 | 35 |
| 30 to 40 | 28 |
| 40 to 50 | 34 |
| 50 to 60 | 17 |
| 60 to 70 | 17 |
| 70 to 80 | 8 |
| 80 to 90 | 1 |
| 90 to 100 | 3 |
| Unknown | 1 |
| Total, | 372 |

| | | | | | |
|---|---|---|---|---|---|
| Males, | 195 | Females, | 177 | Total, | 372 |
| Married, | 112 | Single, | 260 | Total, | 372 |
| White, | 365 | Colored, | 7 | Total, | 372 |

NATIVITIES.

| | |
|---|---|
| Austria, | 1 |
| Bohemia, | 8 |
| Bavaria, | 1 |
| Canada, | 6 |
| Native, Chicago, | 67 |
| Foreign " | 88 |
| U. S., other parts, | 67 |
| Denmark, | 4 |
| England, | 10 |
| France, | 1 |
| Germany, | 40 |
| Holland, | 5 |
| Ireland, | 41 |
| Norway, | 9 |
| Poland, | 1 |
| Switzerland, | 1 |
| Sweden, | 18 |
| Unknown, | 4 |
| Total, | 372 |

MORTALITY BY WARDS FOR THE MONTH.

| Wards. | Mortality. | Pop. in 1868. | One death in |
|---|---|---|---|
| 1 | 6 | 9,094 | 1,515⅔ |
| 2 | 12 | 13,074 | 1,089½ |
| 3 | 16 | 15,076 | 942¼ |
| 4 | 29 | 17,796 | 613⅔ |
| 5 | 19 | 16,033 | 843¾ |
| 6 | 15 | 13,083 | 872 1-5 |
| 7 | 33 | 25,492 | 772½ |
| 8 | 17 | 15,813 | 930⅙ |
| 9 | 19 | 19,297 | 1,150⅔ |
| 10 | 14 | 12,925 | 923 1-5 |
| 11 | 14 | 14,340 | 1,240 2-7 |
| 12 | 17 | 17,485 | 1,280⅔ |
| 13 | 17 | 11,164 | 656⅔ |
| 14 | 18 | 14,839 | 824 2-5 |
| 15 | 23 | 21,078 | 916 4-7 |
| 16 | 23 | 15,465 | 672⅓ |

| Mortality. | |
|---|---|
| Accidents, | 14 |
| County Hospital, | 14 |
| Home for Friendless, | 4 |
| Hospital Alexian Brothers, | 1 |
| Hospital for Women and Children, | 1 |
| Immigrants, | 25 |
| Mercy Hospital, | 6 |
| Marine Hospital, | 3 |
| St. Joseph Orphan Asylum, | 6 |
| Manslaughter, | 1 |
| Lake Michigan, | 1 |
| River, Chicago, | 2 |
| Suicide, | 1 |
| Huron Street Police Station, | 1 |
| Total, | 372 |

The increase of mortality is quite marked compared with the month of May, 1868; and is not to be wondered at, when the extremes of temperature and the great amount of rain that has fallen are borne in mind. More deaths resulted from accidental causes this year than last, whilst the mortality by brain diseases is less. The increase, however, by pulmonary and bowel affections is decided, being 38 more. Owing to the comparative immunity from contagious diseases, the climatic influences on life are still more marked, and will be better appreciated by the following comparative table:—

| | *1868.* | *1869.* | | |
|---|---|---|---|---|
| Highest temperature, | 75° | 81° | + | 6° |
| Lowest " | 43° | 42° | = | 1° |
| Range " | 32° | 39° | + | 7° |
| Mean " | 55° | 54° | = | 1° |
| Rain, - - - | 3748 in. | 5670 | + | 1922 in. |

The number of still and premature births reported this month are about the same as last year, due allowance being made for the increase of population.

---

Pruritus Vulvæ.—It is stated (*Gazette des Hopitaux*, Jan. 7th, 1869) that this annoying affection is often entirely cured and always diminished by a lotion consisting of five parts of corrosive sublimate dissolved in fifty parts of alcohol. A teaspoonful of this solution is to be diluted with a pint of tepid water, and applied as a wash to the parts several times in the day.—*Boston. Med. and Surg. Journal.*

---

There is a doctor for every 1000 inhabitants in Paris, and the apothecaries number 547. There are also 295 officers of health.—*Med. and Surg. Reporter.*

